# ^1^H, ^13^C, ^15^N backbone assignment of the minimally tied trefoil knot, MTT_SA_, a 23s rRNA SPOUT methyltransferase

**DOI:** 10.1007/s12104-026-10262-9

**Published:** 2026-04-10

**Authors:** Tiange Tao, Dominique T. Capraro, Patricia A. Jennings

**Affiliations:** Department of Chemistry and Biochemistry, University of California, La Jolla, San Diego, CA USA

**Keywords:** Methyltransferase, Knotted protein, SPOUT, Protein NMR, MTT (minimally tied trefoil)

## Abstract

Knotted Methyltransferases (MTase) from the SpoU-TrmD (SPOUT) family offer a unique opportunity to study a protein knot topology and enzymatic function. The knotted methyltransferase from *Staphylococcus aureus*, MTT_SA_ (PDB: 1vh0, 4fak), is an ɑ/β-knotted 23s rRNA MTase containing only the minimal scaffold among the SPOUT family members. This dimeric enzyme is a minimalist model to study the deep + 3_1_ knot. Here, we report the non-proline backbone assignments with 98.8% completion using TROSY-based NMR experiments on uniformly labeled ^2^H/^13^C/^15^N-labeled protein. Complementary HBHA(CO)NH experiments on ^13^C/^15^N-labeled protein were completed for Hα/Hβ sidechain assignments. Both backbone and sidechain assignments were deposited in the Biological Magnetic Resonance Bank (BMRB ID: 53391). Of 164 assigned residues, TALOS-N predictions provided 88.4% unambiguous assignments based on the H_N_, H_α_, C_α_, C_β_ and N chemical shifts. In addition, the predicted secondary structure based on torsion angles and the ΔδC_α_-ΔδC_β_ profile are in agreement with the crystal structures.

**Supplementary Information:**

The online version contains supplementary material available at 10.1007/s12104-026-10262-9.

## Biological context

Knotted polypeptide chains represent a rare and complex topological feature among protein folds. With an increasing number of knotted proteins being identified across diverse structural families, understanding their unique topology provides insight into folding, stability, and enzymatic function of the knotted region as these proteins have become increasingly important (Capraro and Jennings 2016). The folding mechanisms of knotted SPOUT MTase has been investigated through computational modeling and characterized with energy landscape theory (Andrews et al. 2013; Burban et al. 2015; Burban and Jennings 2017; Capraro et al. 2020; Dahlstrom et al. 2022; Tkaczuk et al. 2007). Importantly, the correct chirality of the crossing loop is required to overcome the first energy barrier, forming the intermediate slipknot or plug topology. Subsequentially, the C-terminus threads through the loop to establish the native fold of a trefoil knot, which overcomes the second energy barrier (Noel et al. 2010; Strassler et al. 2022; Tkaczuk et al. 2007). Furthermore, matrix map analyses indicate that the active sites of SPOUT enzymes typically reside within the knotted region, where increased intra-chain contacts enhance structural stability and shield bound ligands from solvent accessibility (Dabrowski-Tumanski et al. 2016). Despite these computational and theoretical insights, experimental data at the residue level resolution of rRNA MTases remain limited (Andrews et al. 2013; Burban et al. 2015; Burban and Jennings 2017; Capraro et al. 2020). Solution NMR spectroscopy has provided powerful insight, addressing this gap in knowledge (Burban and Jennings 2017; Capraro et al. 2020). The residue-specific information has generated details regarding backbone conformation, dynamics, and ligand interactions that cannot be fully captured by modeling and crystallography alone (Burban et al. 2015; Burban and Jennings 2017; Capraro et al. 2020; Zhong et al. 2019).

In this paper, we focus on a member of the SPOUT methyltransferase superfamily exhibiting a characteristic deep trefoil (+ 3_1_) knot that contributes to both structural integrity and enzymatic activity. *Staphylococcus aureus*, MTT_SA_ (PDB code: 1vh0, 4fak), is a dimeric 23s rRNA methyltransferase and represents another member of the minimalist model within this knotted protein family (Andrews et al. 2013; Badger et al. 2005; Boundy et al. 2013; Burban et al. 2015; Burban and Jennings 2017; Capraro et al. 2020; Capraro and Jennings 2016; Strassler et al. 2022; Sunita et al. 2008; Tkaczuk et al. 2007). Different from other SPOUT members such as TrmD and RlmB, which incorporate additional secondary structure elements around the knotted core, MTT_SA_ retains only the minimal catalytic architecture, making it a simplified model for investigating the structural and functional role of knots within proteins (Tkaczuk et al. 2007). MTT_SA_ adopts a highly conserved α/β knotted fold characteristic of the SPOUT family, with five β-strands surrounded by five α-helices. The + 3_1_ trefoil knot region is near the C-terminus and is defined by the crossing (T74-L85), wing (D104-L127) and threading loops (S128-F134).

NMR studies of MTT_SA_ enable direct characterization of how knot topology shapes folding, stability, and enzymatic function. Backbone and sidechain resonance assignments serve as an essential foundation for further analyses on many unresolved questions including if the trefoil knot can be fully untied and what additional role the trefoil knot may serve in enzymatic activities. Here, we report the non-proline backbone assignments and H_α_ H_β_ sidechain assignments for the 39 kDa native homodimeric SPOUT MTase, MTT_SA_. TROSY NMR was applied to acquire double and triple resonance NMR spectra on ^2^H/^13^C/^15^N labeled sample to enhance signals for backbone assignment, and the HBHA(CO)NH spectra on ^13^C/^15^N labeled sample were acquired for the sidechain assignment (Fernandez 2003; Foster et al. 2007; Kay and Gardner 1997; Li and Byrd 2022; Pervushin et al. 1998; Xu and Matthews 2011).

## Methods and experiments

### Protein expression and purification

The gene encoding MTT_SA_ was cloned into a pET-24d vector and expressed in *Escherichia coli* BL21(DE3) competent cells. Uniformly ^2^H/^13^C/^15^N and ^13^C/^15^N labeled samples were prepared in D_2_O-M9 minimal medium, with ^15^NH_4_Cl at a concentration of 1.1 g per liter and D-Glucose (U-^13^C_6_) at a concentration 2.0 g per liter. For ^2^H/^13^C/^15^N labeled sample, we used deuterated D-Glucose (U-^13^C_6_, 1,2,3,4,5,6,6-D_7_) at a concentration 2.0 g per liter. MTT_SA_ expression included 50 mg/mL kanamycin as antibiotic, grown at 37 °C until OD_600_ reached 0.6 and was induced with 1mM Isopropyl-β-D-thiogalactopyranoside (IPTG) at 25 °C for 16–18 h. The harvested cells were lysed by sonication in 50mM NaPO_4_, 300mM NaCl, pH 8.0, and the protein was isolated using Ni-NTA (Qiagen) resin with increasing concentration of imidazole. The protein of interest was eluted using 50mM NaPO_4_, 300mM NaCl, 150mM imidazole, pH 8.0. The protein was further purified by size-exclusion chromatography on Superdex 200 in 75mM NaOAc, pH 5.6, 1% glycerol.

### NMR sample preparation

For backbone assignments, purified ^2^H, ^13^C, ^15^N labeled MTT_SA_ was exchanged into 75mM NaOAc-d3, pH 5.6, 1% Glycerol, and 10% D_2_O. Protein was purified in H_2_O media, and sample was allowed to equilibrate in protonated buffer until all NH protons were visible in the ^1^H/^15^N HSQC spectra. TROSY based triple resonance NMR spectra were acquired at 305 K. For sidechain assignments, additional prepared and purified ^13^C, ^15^N labeled MTT_SA_ was exchanged into the same buffer conditions, and the HBHA(CO)NH spectra was acquired at 305 K.

### NMR experiments

All NMR experiments for MTT_SA_ backbone assignments were performed on a Bruker Avance 800 MHz Neo spectrometer equipped with Topspin 4.0.9. 5 mm TXO cryoprobe, with ^15^N optimization. The following TROSY double and triple resonance experiments were conducted to obtain resonance information for the backbone assignments: TROSY-HSQC, TROSY-HNCACB, TROSY-HNCO, TROSY-HNCACO (Fitzkee and Bax 2010). Additionally, the HBHA(CO)NH experiment was performed on a Bruker Avance Neo600 MHz spectrometer in order to obtain side chain proton resonance, enhancing the completeness of MTT_SA_ backbone assignments.

All NMR datasets were acquired through Bruker TopSpin^™^ NMR Software (Version 4.1.4) (Bruker BioSpin). Raw spectral data was processed using NMRfx Analyst (Norris et al. 2016). Resonance assignments were carried out using Sparky (Lee et al. 2015). The backbone assignments were determined based on triple-resonance correlations (Toshio et al. 1994).

### Secondary structure analysis

The secondary structure was evaluated experimentally using the ΔCα-ΔCβ profile (Wishart and Sykes 1994). In α-helical regions, ΔCα is positive and ΔCβ is negative; in β-strands ΔCα is negative and ΔCβ is positive; a value near zero indicates coil. ΔCα-ΔCβ values were calculated from backbone chemical shifts using random coil index as reference (δCα_observed_ - δCα_RCI_) − (δCβ_Observed_ - δCβ_RCI_) (Berjanskii and Wishart 2005). This profile shows regions of positive values consistent with α-helices and negative values consistent with β-strands and interprets contiguous regions of ≥ 3 residues that exceed ± 1ppm. Glycine without Cβ is only assessed with Δ δCα.

### Extent of assignment and data deposition

**Fig. 1:**
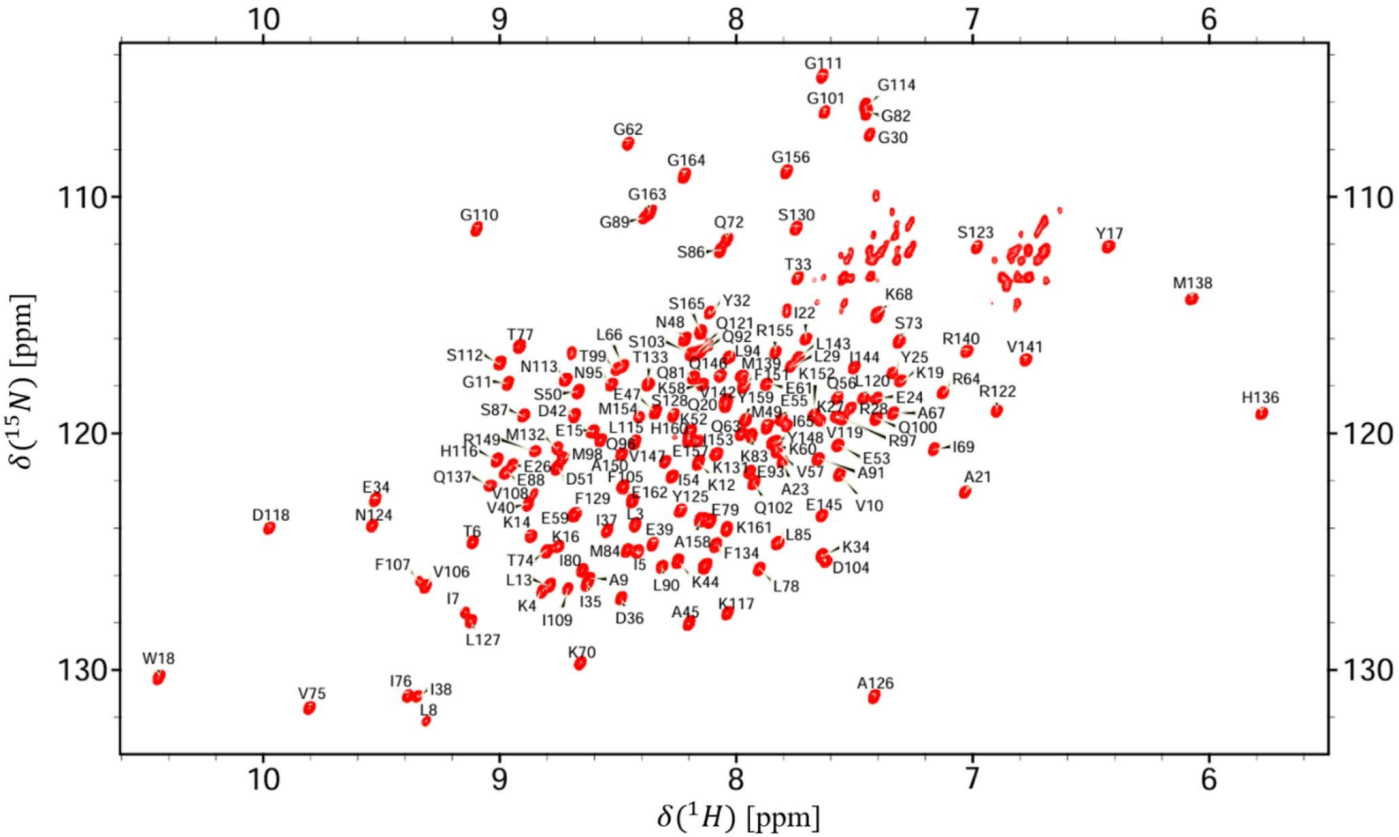
Assigned 800 MHz TROSY-^1^-^15^ N HSQC spectrum of MTT_SA_. Non-proline backbone assignments of uniformly ^2^H/^13^C/^15^N labeled MTT_SA_ were derived from the TROSY-based triple resonance experiments: HNCACB, HNCA, and HNCOCA

Here, we report the ^1^H, ^13^C, ^15^N backbone assignment of MTT_SA_, a 39 kDa dimeric SPOUT MTase. Chemical shift assignments were deposited in the BMRB, ID 53,391. Figure 1 represents the assigned ^1^-^15^ N TROSY HSQC spectrum. NMR data is visualized and assigned as one protomer. All backbone and sidechain chemical shifts were determined through a series of 3D NMR experiments (see methods) by tracing the previous residue chemical shifts.

**Fig. 2:**
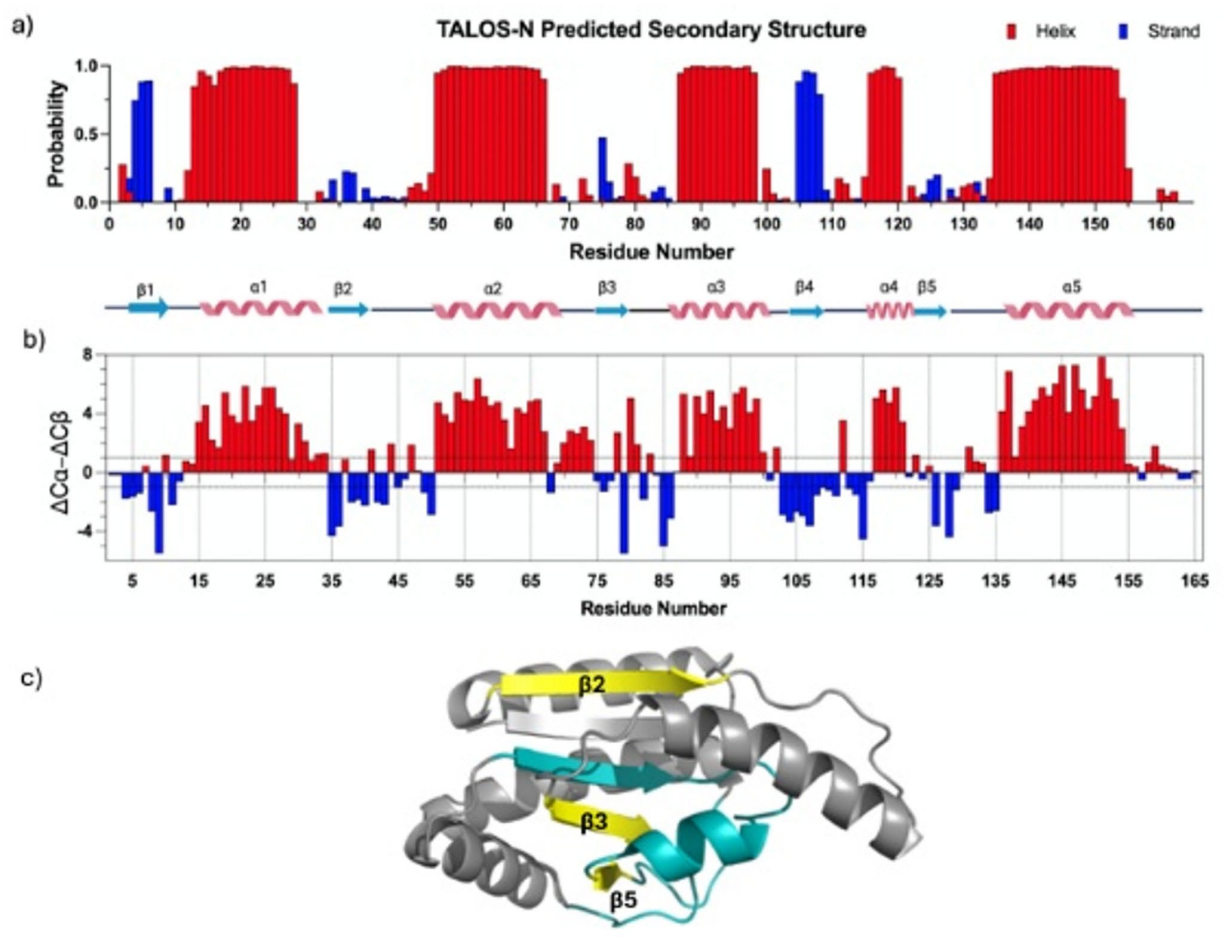
(a) TALOS-N secondary structure prediction comparison with crystal structure (b) Bar plot of secondary chemical shift analysis (ΔCα-ΔCβ) of each residue. Secondary chemical shifts were computed as (Observed – Random Coil Index). Differences (ΔCα-ΔCβ) less than − 1ppm are characterized as β-strand while differences greater than 1ppm are characterized as α-helical. (c) Monomer structure of MTT_SA_, β2, β3 and β5 are highlighted in yellow and ligand binding region is colored in cyan

Assignments for H_α_ and H_β_ were obtained from the HBHA(CO)NH experiments to further complement and complete the backbone details. Backbone assignments excluded prolines (Pro31, Pro41, Pro46, Pro71, Pro135), N-terminus initiator (Met1) and C-terminus Histidine-tag (His166-171). The remaining assignments were obtained with 98.8% completion, where residue Ser2 lacked ^1^H_N_/^15^N assignment. 86.1% of ^1^H_α_, 99.4% of ^13^C_α_, 92.1% of ^13^C_β_, and 99.4% of ^13^CO resonances were assigned. ^1^H_β_ resonances were assigned for a subset of residues, but the assignments were not sufficiently complete to provide meaningful statistics. All chemical shifts assignments including H_N_, H_α_, C_α_, C_β_ and N, were submitted to the TALOS-N server (https://spin.niddk.nih.gov/bax/software/TALOS-N/) for secondary structure prediction based on the predicted backbone φ/ψ torsion angles and sidechain χ1 torsion angles (Fig. 2a) (Shen and Bax 2013). Of the 164 assigned residues, TALOS-N classified 81.7% as Good (Strong), and 6.7% as Good (Generous), yielding 88.4% unambiguously assigned coverage. The predicted secondary structure pattern agrees well with the crystal structure (PDB: 1vh0), especially all α-helices with some minor variations of the β-strands. In our ΔCα-ΔCβ profile (Fig. 2b), β2, β3 and β5 show negative values where each align with the corresponding β-strand in the crystal structure. The boundaries from the chemical shifts are in close agreement with the crystal structure. Interestingly, we observed several differences in the length determination of the β-strands. Our solution-NMR data indicated that β2 and β3 are shorter, and the 3-residue strand (β5) is not detected (Fig. 2c). The discrepancies between the NMR and crystal structure are due in part to the short β strands unraveling in the solution dynamics compared with static crystalline model (Eyal et al. 2005; Hinsen 2008). Furthermore, β2, β3 and β5 lie near the ligand-binding pocket (Fig. 2c), a dynamic region where stabilization as a result of ligand-binding is observed Boundy et al. 2013; Wallin et al. 2007). Taken together, the results presented provide the basis for future evaluations of the folding, stability, ligand binding stabilization and enzyme function of MTT_SA_.

## Supplementary Information

Below is the link to the electronic supplementary material.

Supplementary Material 1

## Data Availability

Chemical shift assignments for MTT SA reported in this manuscript are deposited in the Biological Magnetic Resonance Data Bank (BMRB), access ID: 53391. All other data sets are available upon request.
