# Supplementary material for: 1H, 13C, 15N backbone assignment of the minimally tied trefoil knot, MTTSA, a 23s rRNA SPOUT methyltransferase
Source: Biomol NMR Assign. 2026 Apr 10;20(1):16. doi: 10.1007/s12104-026-10262-9 (PMC13068757; doi:10.1007/s12104-026-10262-9)
Supplement: Supplementary file 1 — Supplementary Material 1 [file 12104_2026_10262_MOESM1_ESM.docx]

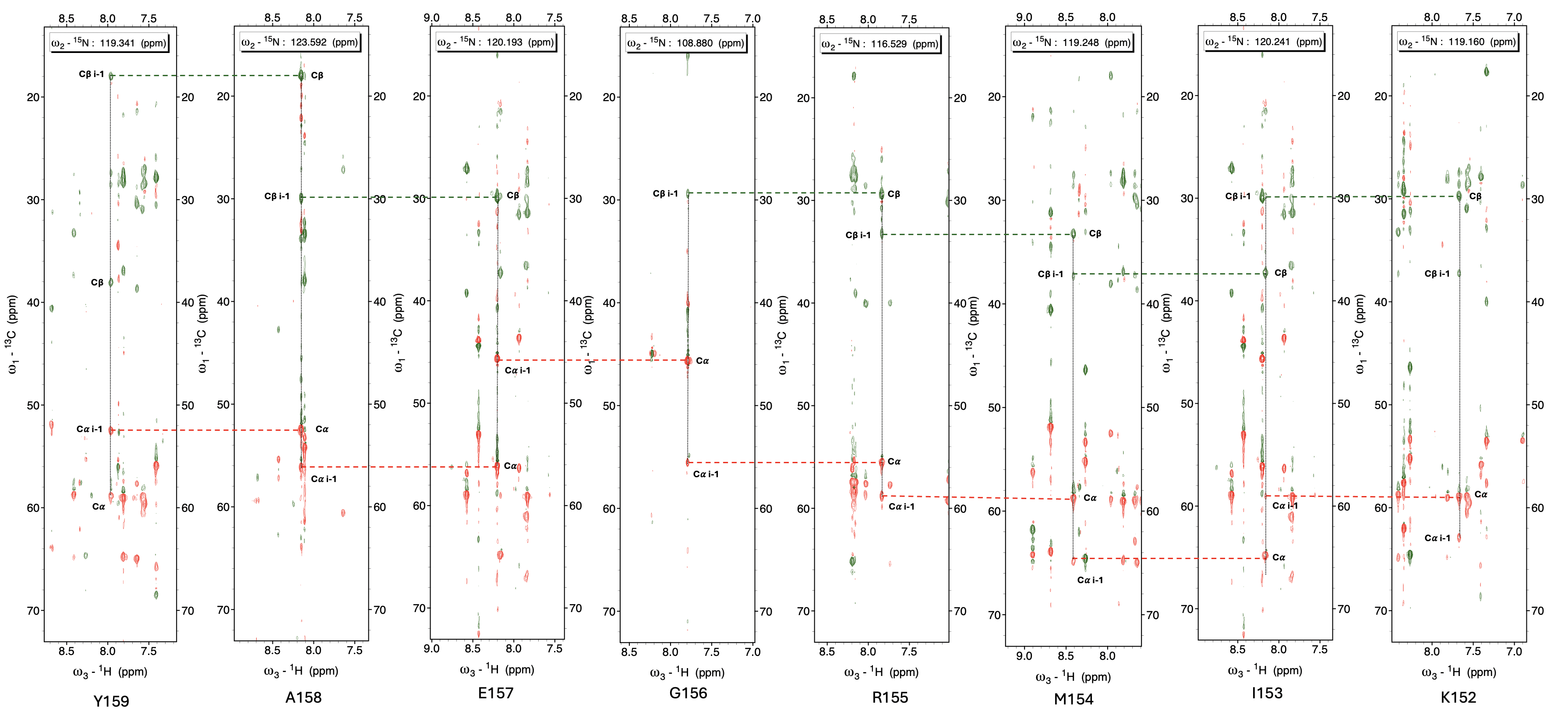


**Supplementary Figure 1**. TROSY-NHCACB spectra stripe plots from K152 to Y159 show backbone assignments strategy of MTT_SA._  Each stripe plot has corresponding H_N_ chemical shifts, in which positive signals are colored red, and negative signals are colored green. The stronger peaks represent the current residue chemical shift (Cα_i_ and Cβ_i_ ), and weaker peaks represent the preceding residue chemical shift (Cα_i-1_ and Cβ_i-1_). Backbone assignments are completed by finding identical Cα/Cβ pairs in another H_N_ group.
